# Unique Complications of MIS-C and Its Treatment: Encephalopathy in a Child with MIS-C Who Developed Life-Threatening Gastrointestinal Hemorrhage

**DOI:** 10.1155/2022/7244434

**Published:** 2022-10-22

**Authors:** Luke Burton, Ananya Manchikalapati, Chrystal Rutledge, Jeremy M. Loberger, Nicholas Rockwell, Joshua Cooper, Maggie Lawrence, William C. Sasser

**Affiliations:** ^1^University of Alabama at Birmingham, Birmingham, AL, USA; ^2^Children's of Alabama, Birmingham, Alabama, USA

## Abstract

In this case report, we describe a previously healthy eleven-year-old male diagnosed with multisystem inflammatory syndrome in children (MIS-C) associated with coronavirus disease 2019. The patient presented with shock and neurologic symptoms including altered mental status and dysarthria. Brain magnetic resonance imaging, obtained to rule out thromboembolic injury, demonstrated cytotoxic edema of the corpus callosum, an imaging finding similar in nature to several previous reports of MRI abnormalities in children with MIS-C. Following administration of intravenous immunoglobulin and pulse-dose steroids, the patient convalesced and was discharged home. Medications prescribed upon discharge included a steroid taper, daily aspirin, and proton pump inhibitor. Four days later, he was readmitted with shock and life-threatening gastrointestinal (GI) hemorrhage. After extensive evaluation of potential bleeding sources, angiography revealed active bleeding from two arterial vessels supplying the duodenum. The patient demonstrated no further signs of bleeding following successful coil embolization of the two vessels. We hypothesize that the vasculitic nature of MIS-C combined with anti-inflammatory and antithrombotic therapy placed him at risk of GI hemorrhage. This case highlights unique radiologic features of MIS-C as well as potential complications of treatment.

## 1. Introduction

Multisystem inflammatory syndrome in children (MIS-C) is a recently described phenomenon associated with coronavirus disease 2019 (COVID-19) [1]. Children typically present with fever and laboratory evidence of systemic inflammation. Additional signs and symptoms can vary widely, leading to diagnostic and management challenges. While gastrointestinal (GI) complaints appear to be the most common symptoms on presentation, general malaise, rash, neurologic complaints, and symptoms related to cardiovascular dysfunction may be present [1]. The spectrum of disease severity in patients with MIS-C also varies. Preliminary epidemiologic data suggests that the majority of children hospitalized with MIS-C require intensive care, commonly for management of shock and/or respiratory failure [1, 2].

Given the range of clinical manifestations in children with MIS-C, it is important to report unique cases that represent uncommon but life-threatening complications associated with the disease and its management. We report the case of a child diagnosed with MIS-C who initially presented with shock and encephalopathy followed by a second admission with severe upper GI hemorrhage. The patient's brain magnetic resonance imaging (MRI) demonstrated inflammation of the corpus callosum, an imaging finding similar in nature to those in recent reports of fifteen children with COVID-19 and/or MIS-C and neurologic dysfunction [3–7]. We hypothesize that the vasculitic nature of MIS-C combined with anti-inflammatory treatment caused severe GI bleeding. To our knowledge, this is the first report of a child with life-threatening GI hemorrhage in the setting of MIS-C.

## 2. Case Report

In late November 2020, a previously healthy eleven-year-old male presented to an emergency department with a 2-day history of fever (maximum temperature 104°Fahrenheit), malaise, and confusion. He was in uncompensated shock, prompting fluid resuscitation, initiation of inotropic support, and admission to the Pediatric Intensive Care Unit (PICU) for ongoing care. Laboratory evaluation revealed a hyperinflammatory state with elevated C-reactive protein, procalcitonin, erythrocyte sedimentation rate, and ferritin (Table 1). Echocardiogram revealed mildly diminished left ventricular systolic function and normal coronary artery anatomy with elevated troponin and brain natriuretic peptide (Table 1). His neurologic symptoms, including agitation, dysarthria, and ultimately aphasia, prompted brain MRI that demonstrated cytotoxic edema involving the corpus callosum and restricted diffusion in portions of the bilateral centrum semiovale and internal capsules. He tested positive for COVID-19 by nucleic acid amplification and had serologic evidence of SARS-CoV-2 IgG antibodies. A presumptive diagnosis of MIS-C was made. Lumbar puncture was not performed.

Treatment focused on inotropic support and anti-inflammatory therapy, including 1 dose of intravenous immunoglobulin (IVIG) (2 g/kg) and 3 days of pulse-dose methylprednisolone (23 mg/kg/dose, equivalent to 1 g/dose). Prophylactic low-molecular weight heparin was initiated due to relative immobility and concern for hypercoagulability associated with MIS-C. His shock and mental status improved over the course of the next forty-eight hours. He was discharged home 3 days later following normalization of mental status and cardiac function. Upon discharge, he was continued on an oral steroid taper and placed on aspirin (ASA) (81 mg/day) and an oral proton pump inhibitor (PPI).

Four days after discharge, the patient returned to the emergency department in hemorrhagic shock. His parents reported that within the prior twenty-four hours, the patient experienced progressive dizziness and had a melanotic stool. Laboratory evaluation revealed a hemoglobin of 2.6 g/dL and profound metabolic acidosis with elevated serum lactate in the presence of a normal platelet count (Table 1). Exam was notable for significant pallor and sleepiness. He was resuscitated with blood products with resultant stabilization of hemodynamics and improvement in mental status. Computer tomography (CT) scan of his abdomen and pelvis demonstrated colonic and small bowel wall thickening. He was admitted to the PICU for ongoing care. Given suspicion for GI bleed, an intravenous PPI was scheduled and octreotide infusion initiated.

Following 8 hours of hemodynamic stability, the patient acutely developed altered mental status and hypotension and passed a large volume of bright red blood per rectum. Treatment included intubation and mechanical ventilation, aggressive blood product resuscitation (79 mL/kg packed red blood cells, 26 mL/kg platelets, and 17 mL/kg of fresh frozen plasma), administration of recombinant factor VII (90 mcg/kg), and initiation of an aminocaproic acid infusion. Neither CT angiogram nor upper and lower endoscopies revealed a clear source of active bleeding. After a third episode of hemodynamic instability associated with large-volume GI tract blood loss, interventional radiology performed an angiogram that demonstrated two areas of active bleeding in the duodenal region. Branch vessels of the gastroduodenal and pancreaticoduodenal arteries associated with the bleeding were successfully coil embolized. Following the procedure, the patient was extubated and demonstrated no further clinical or laboratory evidence of bleeding. He was discharged home in a stable condition following several days of observation. His only medication at discharge was an oral PPI.

## 3. Discussion

We describe the case of an eleven-year-old male with MIS-C who experienced shock, encephalopathy, and life-threatening GI bleed. This case highlights unique neurologic manifestations of MIS-C and potential side effects of pharmacologic treatment.

### 3.1. Neurologic Symptoms Associated with MIS-C

Between 20 and 34% of children with MIS-C experience neurologic symptoms [7, 8]. Headache, altered mental status, and encephalopathy are among the most frequently described neurologic symptoms [4, 9–11]. The pathophysiology of neurologic injury in MIS-C is not fully understood. Mechanistic theories include direct viral injury to neural cells, vascular endothelial cell-mediated injury, and injury related to dysregulated autoimmune response [8, 10].

### 3.2. Neurologic Symptoms with Characteristic MRI Findings in This Patient

Our patient's first admission was notable for neurologic symptoms including encephalopathy, dysarthria, and ultimately aphasia. These symptoms raised concern for thromboembolic stroke, prompting performance of a brain MRI. The MRI demonstrated no signs of stroke but rather extensive cytotoxic edema of the corpus callosum and restricted diffusion in portions of the bilateral centrum semiovale and internal capsules. Cytotoxic edema of the corpus callosum is thought to occur secondary to dysregulated immune responses to a variety of stimuli including drugs, trauma, subarachnoid hemorrhage, Kawasaki disease (KD), and hemolytic uremic syndrome [12, 13]. The corpus callosum, which has a particularly high concentration of glutamate receptors, is predisposed to intramyelin edema related to cytokine-mediated glutamate release [3, 5]. Clinical manifestations of diseases affecting the corpus callosum, particularly the posterior portion or splenium, include confusion, ataxia, dysarthria, seizures, and headache [14]. Fortunately, these lesions and associated clinical symptoms are typically reversible [13]. A clinical-radiologic syndrome known as cytotoxic lesion(s) of the corpus callosum, radiographically characterized by variable-sized lesions predominantly located in the splenium, has been reported in twenty children diagnosed with COVID-19 and/or MIS-C, supporting potential mechanistic link to the diseases [3–8].

### 3.3. Gastrointestinal Symptoms Associated with MIS-C

GI symptoms are common in patients with MIS-C [1]. In a series of forty-four patients with MIS-C admitted to Children's Hospital at Columbia University Irving Medical Center, Miller et al. evaluated the frequency and nature of GI symptoms. They found that 84% of patients reported GI symptoms on admission, with 75% reporting abdominal pain, 57% vomiting, and 40% diarrhea. Two patients experienced hematochezia or melanotic stools. Imaging findings included mesenteric adenitis (2 patients) and bowel wall thickening (3 patients) [15]. These radiologic findings may lead to diagnostic challenges given clinical overlap with conditions like appendicitis and inflammatory bowel disease.

### 3.4. Gastrointestinal Hemorrhage in This Patient

To our knowledge, this is the first report of hemodynamically significant gastrointestinal hemorrhage in a patient with MIS-C. On readmission with hemorrhagic shock, concern for a bleeding gastric ulcer was high given recent steroid and ASA use. Demonstration of bowel wall thickening on CT scan also raised concern for enteritis and/or colitis-associated bleeding. Inability to pinpoint a source of bleeding with CT and endoscopy prompted angiographic evaluation. This revealed two areas of bleeding in the duodenal region amendable to coil embolization. Cause of vessel disruption remains unclear. Because the duodenum was not completely visualized endoscopically, it is possible that an undiagnosed duodenal ulcer precipitated the hemorrhage.

It is also possible that vascular inflammation associated with MIS-C contributed to the patient's GI hemorrhage. A subset of patients with MIS-C share clinical characteristics with KD, a medium vessel vasculitis. This clinical overlap suggests that postinfectious vasculitis may play a role in the pathogenesis of MIS-C. A recent study evaluating hematologic characteristics and cytokine profiles in children with COVID-19 and MIS-C demonstrated evidence of endothelial disruption in patients with MIS-C [16]. The role of vascular inflammation as it relates to GI symptomology in MIS-C remains largely unknown.

### 3.5. Treatment Considerations

It is important to acknowledge the role pharmacologic treatment may have played in the GI hemorrhage. Anti-inflammatory therapy for our patient included IVIG and pulse-dose steroids. The American College of Rheumatology's (ACR) recently published guideline for management of children with MIS-C suggests a tiered approach to immunomodulatory therapy based on degree of illness. Recommendations include consideration of high-dose IVIG (1-2 g/kg) and low-to-moderate-dose steroids (1-2 g/kg). Pulse-dose steroids (10-30 mL/kg/day), as our patient received, may be considered when life-threatening complications accompany MIS-C diagnoses [17].

Regarding antiplatelet therapy, the ACR recommends the use of ASA for patients with MIS-C and laboratory evidence of thrombocytosis and presence of coronary artery aneurysms and in those with features of KD [17]. Our patient was initially treated with low-molecular-weight heparin and transitioned to ASA prior to discharge. He was prescribed a PPI while inpatient and upon both hospital discharges. Steroid and anti-thrombotic therapy, while clinically indicated, may have increased risk of GI hemorrhage. It is important to note the prolonged antiplatelet effect of ASA. Clinically, this becomes significant in patients with severe bleeding as platelet administration is necessary to combat ASA's prolonged effect despite normal platelet counts.

## 4. Conclusions

This case describes a critically ill child who experienced two unique complications associated with MIS-C and its treatment, highlighting diagnostic and treatment dilemmas. We hope its review will provide guidance for clinicians caring for children with MIS-C with associated neurologic and GI symptoms.

## Figures and Tables

**Table 1:** Lab results for the patient are shown.

Parameters	Day 1 of initial presentation	Day 1 of readmission	Day 2 of readmission	Reference ranges
WBC (10^3^/*μ*L)	16.56	56.2	19.2	4.31-11.00
Hemoglobin (g/dL)	9.7	2.6	7.8	10.7-13.4
Hematocrit (%)	28.2	9.2	22.9	32.3-39.8
Platelets (10^3^/*μ*L)	111	369	48	140-440
C-reactive protein (mg/dL)	20.1	0.19		0.00-0.50
Procalcitonin (ng/mL)	27.02	0.28		0.00-2.00
Lactic acid (mmol/L)	3.1	19.7	4.8	0.7-2.1
ESR (mm/h)	70	124		(0-15)
Ferritin (ng/mL)	1970			13.7-78.8
PT (second)	16	20.5	11.4	12.1-14.6
aPTT (second)	38.7	22.3	33	23.5-36.4
INR	1.3	1.8	0.9	0.9-1.2
Troponin (ng/mL)	1.83			Normal: <0.05 ng/mL Indeterminate: 0.05-0.39 ng/mL Highly suggestive of cardiac muscle damage: ≥0.40 ng/mL
Brain natriuretic peptide (pg/mL)	2978			7.0-21.0 pg/mL
SARS-CoV-2 antibodies, IgG	Positive	Positive		
COVID-19 by nucleic acid amplification	Positive	Negative		

WBC: white blood cell; ESR: erythrocyte sedimentation rate; Ig: immunoglobulin.

## Abbreviations

MIS-C: Multisystem inflammatory syndrome in children
GI: Gastrointestinal
COVID-19: Coronavirus disease 2019
MRI: Magnetic resonance imaging
PICU: Pediatric Intensive Care Unit
IVIG: Intravenous immunoglobulin
ASA: Aspirin
PPI: Proton pump inhibitor
CT: Computer tomography
KD: Kawasaki disease.
